# Magnetic, Fluorescent, and Copolymeric Silicone Microspheres

**DOI:** 10.1002/advs.201500114

**Published:** 2015-05-05

**Authors:** Jacqueline M. Rankin, Nitin K. Neelakantan, Kimberly E. Lundberg, Elissa M. Grzincic, Catherine J. Murphy, Kenneth S. Suslick

**Affiliations:** ^1^ Department of ChemistryUniversity of Illinois at Urbana‐Champaign600 S. Matthews AveUrbanaIL61801USA

**Keywords:** aerosol polymerization, drug delivery, microencapsulation, polydimethylsiloxane, sonochemistry

## Abstract

**Silicone microspheres are exceedingly difficult to make**. Here, polydimethylsiloxane microspheres (≈1 μm diameter) are synthesized using ultrasonic spray pyrolysis, the first demonstration of a scalable synthetic procedure for crosslinked silicone microspheres. This continuous, aerosol process is also used to directly produce fluorescent, magnetic, and copolymeric derivatives; the potential biomedical applications of these microspheres are explored.

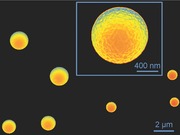

Silicones, polymers with a backbone of silicon**–**oxygen bonds, are widely used in the fields of chemistry and materials science. Of the silicone family, polydimethylsiloxane (PDMS) is the most commonly employed. The unique rheological properties, low glass transition temperature, optical transparency, temperature stability, high chemical resistance, biocompatibility/low toxicity, high gas permeability, and hydrophobicity of PDMS have made PDMS the material of choice for everything from gas chromatography stationary phase and microextraction materials to additives in shampoos, food, and lubricating oils to contact lenses, medical devices, and implants.^1^, ^2^, ^3^ Surprisingly, only a handful of reports of microspheres made from PDMS exist in the literature^4^, ^5^, ^6^, ^7^, ^8^, ^9^, ^10^, ^11^, ^12^ and traditional emulsion polymerizations of silicone spheres produce large, polydisperse microspheres. This is due, in large part, to the high viscosity and low surface energy of PDMS oligomers, which cause coalescence and aggregation during emulsion polymerizations, especially at the high temperatures necessary for polymer curing.^10^, ^13^, ^14^

Despite their difficult fabrication, many potential applications for PDMS microspheres have been suggested in the literature. Proposed uses include sensors,^5^, ^15^, ^16^ actuators,^8^ and additives for polymer resins.^7^, ^10^, ^11^ PDMS microspheres have also been suggested as materials for extraction and chromatography^11^, ^12^ and biomedical applications including drug delivery and controlled release.^6^, ^9^, ^10^, ^11^ These applications have not yet been well explored, arguably due to the lack of versatility and control inherent in past PDMS microsphere syntheses. Ultrasonic spray pyrolysis (USP) has previously been utilized to make microspheres of various materials, including porous silica,^17^ porous carbon,^18^, ^19^ metal oxides and composites,^20^, ^21^ and metal sulphides.^22^, ^23^ USP is a one‐step, continuous, aerosol process that produces microspheres that are generally micrometers in diameter with relatively narrow size distributions. We report here the fabrication of PDMS microspheres using USP. This approach is advantageous over the more common PDMS microsphere synthetic methods; each droplet acts as its own isolated microreactor, reducing the chance of prepolymer coalescence and aggregation. Additionally, the size and composition of the resultant product are easily adjusted by altering the concentration and choice of precursors in the nebulized solution.

The USP preparation of PDMS microspheres uses a simple ultrasonic transducer (1.7 MHz) to generate a mist from a precursor solution that is then swept by an inert gas flow through a heated tube (illustrated in Figure S1 in the Supporting Information). A solution of PDMS (Sylgard 184; 2:1 base to accelerator) in hexanes was nebulized using this apparatus and the aerosol carried through a furnace set at 300 °C via an Ar stream (0.4 slpm). The resulting product is collected in EtOH bubblers, washed three times with EtOH (using an ultrasonic bath) followed by centrifugation, and resuspended in hexanes. SEM (**Figure** **1**a) of the product shows well‐formed microspheres with minimal agglomeration. FTIR of the dried product (Figure 1b) matches the IR spectrum of PDMS reported in the literature,^10^, ^24^ and Raman mapping (Figure 1c) confirms the PDMS signal originates from the microspheres and not from any residual unreacted PDMS or nonspherical cross‐linked PDMS. Thermogravimetric analysis (Figure S2, Supporting Information) is consistent with that of bulk PDMS.^25^

**Figure 1:**
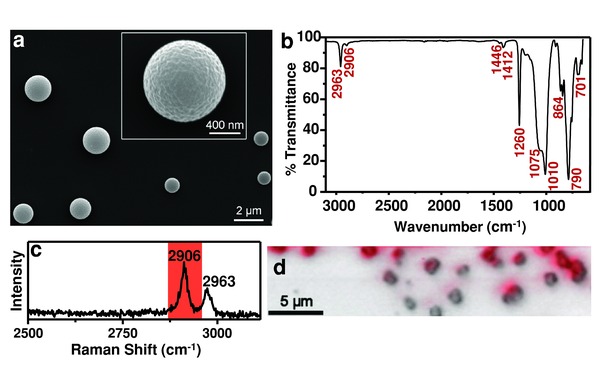
Polydimethylsiloxane (PDMS) microspheres prepared using ultrasonic spray pyrolysis. a) SEM of microspheres. The inset shows expanded view of microsphere. b) ATR‐FTIR spectrum of resulting product; peaks match literature values for PDMS.^10^, ^24^ c) Raman spectrum of product, C—H stretching peaks (2906 and 2963 cm^−1^) for PDMS are clearly evident.^26^ d) Optical image of PDMS microspheres overlaid with Raman mapping showing the relative intensity of the C—H stretching peak (2906 cm^−1^, highlighted in (c)) as the intensity of red coloration.

The average particle size can be tuned by adjusting the concentration of PDMS in the precursor solution (**Figure** **2**). Nebulization of a 20 mg mL^−1^ precursor solution produced microspheres with an average diameter of 1.1 μm (Figure 2c,d). Reducing the concentration of PDMS in the precursor solution to 4 mg mL^−1^ reduces the microspheres' average diameter to 890 nm (Figure 2a,b), while increasing the concentration of PDMS in the precursor solution to 100 mg mL^−1^ increases the average diameter to 2.0 μm (Figure 2e,f). In all cases, the relative standard deviation is ≈30%. Increasing the concentration of PDMS to >100 mg mL^−1^, however, produces a precursor solution too viscous to nebulize. We have made microspheres with average diameters as small as ≈500 nm, simply by further reducing the concentration of PDMS in the precursor solution (Figure S3, Supporting Information). We expect the average microsphere diameter could be reduced even further by nebulizing more dilute precursor solutions; as shown in Figure S3 (Supporting Information), microspheres whose diameters are as low as ≈100 nm are observed.

**Figure 2:**
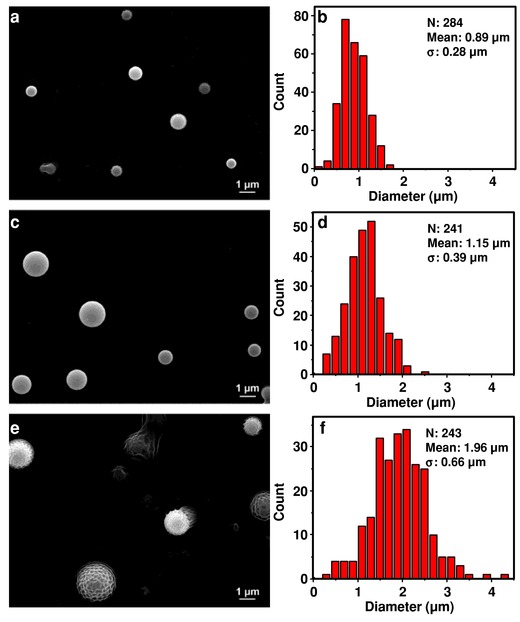
Size control of polydimethylsiloxane (PDMS) microspheres. a,c,e) SEMs of PDMS microspheres obtained with a) 4 mg mL^−1^, c) 20 mg mL^−1^, and e) 100 mg mL^−1^ PDMS in hexanes. b,d,f) Size distributions of microspheres shown in (a,c,e), respectively. Size distributions were determined using the Image J software package, with total number of microspheres counted given as *N*.

The USP preparation of silicone microspheres produces particles with average diameters <2 μm and narrow size distributions; because each precursor droplet acts as its own isolated microreactor, the chance of prepolymer coalescence and aggregation is reduced, and the resultant microspheres have much smaller diameters and narrower size distributions as compared to products obtained using conventional synthetic methods. In contrast, due to the very low surface energy of silicones, emulsion polymerizations of silicone spheres (e.g., using vortex shakers or mechanical stirrers) produce large, polydisperse microspheres with diameters in the 50 to hundreds of μm range and multimodal distributions.^6^, ^10^, ^11^, ^27^ Other synthetic methods include the synthesis of liquid PDMS microspheres by rapid expansion of a supercritical solution,^5^ synthesis of crosslinked PDMS magnetic microspheres using a microfluidic channel,^8^ formation of PDMS microparticles via grinding silicone tubing under liquid nitrogen,^12^ and a “one‐at‐a‐time” synthesis of PDMS microspheres utilizing a fiber dipped into noncrosslinked PDMS.^15^ These methods are cumbersome, have low production rates, produce only large microspheres (>100 μm to 1 mm in diameter), and have therefore gained little traction. The USP synthetic method described here overcomes all of these limitations; the process is simple, continuous, scalable, and can easily be adjusted to produce microspheres with diameters in the hundreds of nanometer to few micrometer range.

Magnetic PDMS microspheres would be interesting materials for MRI contrast agents, hyperthermia therapy, and targeted drug delivery. We have successfully formed core‐shell magnetic PDMS microspheres by simply adding a commercially available colloidal suspension of 10 nm Fe_3_O_4_ nanoparticles (2% v/v Magna View Fluid, United Nuclear) to a 20 mg mL^−1^ silicone precursor solution. After USP under the same conditions, the resulting product was vacuum filtered through a 0.22 μm Teflon filter, washed with three aliquots of 50 mL hexanes, and resuspended in hexanes. The product was light brown in color and could be pulled from suspension using a magnet (**Figure** **3**a). SEMs of the magnetic microspheres show similar surface topography and microsphere size to the nonmagnetic microspheres (Figure 3b). An EDS line scan (Figure 3c) confirms the presence of both iron and silicon and shows a core‐shell morphology in which an iron‐rich core is surrounded by a ≈200 nm PDMS shell. TEM of the nonmagnetic PDMS microspheres indicates the microspheres are uniform in density and composition throughout (Figure 3d), while TEM of the magnetic product clearly confirms the core‐shell morphology (Figure 3e). While the majority (≈75%) of these magnetic core‐shell microspheres have only a single iron core, there are some microspheres formed with multiple, smaller iron cores; microspheres having as many as six cores were observed (Figure S4, Supporting Information). Additionally, the magnetic core size can easily be altered by adjusting the concentration of the magnetic dopant in the precursor solution (Figure S5a,b, Supporting Information). A slight reduction in average microsphere diameter is observed for the magnetic microspheres compared to nonmagnetic microspheres obtained with the same concentration of PDMS in the precursor solution. Similarly, a slight reduction in average microsphere diameter is observed for the magnetic microspheres with the smaller magnetic core (Figure S5c–e, Supporting Information).

**Figure 3:**
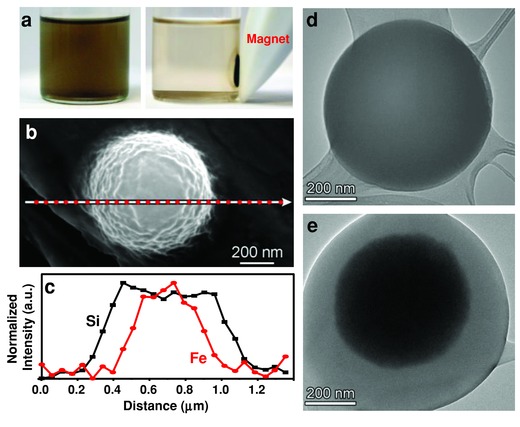
Magnetic core‐shell polydimethylsiloxane (PDMS) microspheres prepared using ultrasonic spray pyrolysis. a) Image of magnetic PDMS microspheres showing (left) microspheres dispersed in hexanes and (right) microspheres pulled from solution using a magnet. b) SEM of magnetic PDMS microsphere on copper foil showing path of energy‐dispersive X‐ray spectral (EDS) line scan. c) EDS line scan showing Si and Fe concentrations along the path line shown in (b). d) TEM of PDMS microsphere (prepared without Fe_3_O_4_) showing uniform density and composition throughout the sphere. e) TEM of magnetic microsphere showing core‐shell structure.

PDMS microspheres doped with a chemically responsive or fluorescent dye have potential applications ranging from sensors to biological imaging to instrument calibration. There are two very different methods to incorporate a dye or fluorophore into these microspheres: one may include the dye in the initial USP synthesis or one may incorporate the dye *after* microsphere synthesis using a swelling solvent. Using Nile red as an example, if one includes the dye in the nebulized precursor solution, the dye cannot be extracted from the resulting microspheres (e.g., into ethanol). In the other method (postsynthetically modifying the microspheres by incorporating Nile red dissolved in chloroform), leaching of the dye from the resulting microspheres will indeed occur in ethanol. We speculate that the presence of the dye during crosslinking creates a doped polymeric structure where the dye is physically trapped within small cross‐linked cavities. In contrast, loading the dye post cross‐linking by using a strongly swelling solvent creates a doped polymeric structure in which the dye is not well confined and can more readily diffuse and leach from the network.

As a proof of concept, we have produced PDMS microspheres doped with the fluorescent dye Nile red using USP. To produce the fluorescent microspheres, Nile red (0.062 m) was added to a 20 mg mL^−1^ precursor solution, and the USP synthesis was performed as described previously. The red colored microspheres were washed with 50 mL EtOH three times, washed with 50 mL hexanes three times, and stored dispersed in hexanes; these microspheres retained fluorescence even after washing and storage for >1 month in ethanol. The diffuse reflectance spectrum (**Figure** **4**a) of the dried final product shows an absorption band at 522 nm that is absent in the nonfluorescent spheres, indicating the successful inclusion of Nile red. Fluorescence images (Figure 4b) of the Nile red doped microspheres show localized fluorescence. These microspheres were subsequently used for cell uptake experiments (Figure S4c,d, Supporting Information). The fluorescent microspheres were isolated by centrifugation, washed with aqueous PBS, resuspended in the culture medium, and incubated with metastatic human breast cancer cells (MDA‐MB‐231) for 24 h before imaging with confocal fluorescence microscopy. The fluorescent microspheres are clearly evident in both brightfield (Figure 4c) and fluorescence (Figure 4d) images and are localized to the cell cytosol, without further penetration into the cell nucleus. We believe, therefore, that the USP PDMS microspheres are candidates both for biological imaging and potentially for delivery of small molecules into cells.

**Figure 4:**
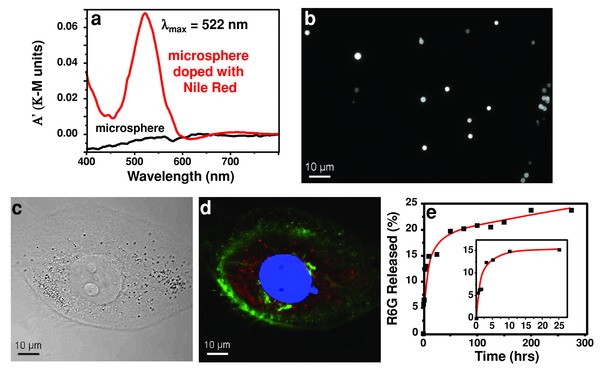
Fluorescent polydimethylsiloxane (PDMS) microspheres, cell uptake, and small molecule release. a) Diffuse reflectance spectrum of Nile red doped PDMS microspheres (red) and nonfluorescent PDMS microspheres (black). Peak at 522 nm indicates successful inclusion of Nile red. b) Fluorescence image of Nile red doped PDMS microspheres. c) Bright field image of cell that has taken up microspheres. d) Fluorescent image of cell in (c). The cell membrane has been stained green, the cell nucleus has been stained blue, and the Nile red doped microspheres appear red. e) Extended release of a small molecule, Rhodamine 6G, from PDMS microspheres into phosphate buffered saline; release reported as percent R6G released of total loaded R6G.

For biomedical applications, microspheres must have extremely low cytotoxicity. To this end, we have investigated the cytotoxicity of our PDMS microspheres. Using the MDA‐MB‐231 human breast cancer cell line, cellular incubation (for 24 h with the PDMS microspheres under the same conditions used for the uptake experiments) produced extremely high cell viability (99%), even at the highest concentration tested, 10^5^ microspheres per cell (Figure S6a, Supporting Information). No statistical difference was seen among the four concentrations tested and the control; representative fluorescence images with live/dead staining are given in Figure S5 (Supporting Information). This extremely low cytotoxicity is consistent with the excellent biocompatibility exhibited by bulk PDMS, which is the material of choice for many biomedical devices.^3^

The uptake of small hydrophobic molecules by bulk PDMS, including dyes and drugs, is a commonly reported problem for microfluidic applications.^28^, ^29^, ^30^ For drug delivery using microspheres, however, the strong sorption characteristics of PDMS could be advantageous. To that end, we have studied the loading and release of a small hydrophobic molecule, Rhodamine 6G (R6G), as a model to explore the potential of our PDMS microspheres for drug delivery. R6G concentrations are easily quantified using UV–vis (Figure S7, Supporting Information). Additionally, R6G has a partition coefficient similar to many active pharmaceutical agents (Table S1, Supporting Information).^28^, ^31^, ^32^ We successfully loaded our USP PDMS microspheres with R6G and monitored its slow release from the microspheres while suspended in phosphate buffered saline at 37 °C, as demonstrated in Figure 4e (details in the Supporting Information). For these studies, we have loaded R6G after the synthesis of the microspheres. For incorporation of expensive pharmaceutical agents (PA) into an inexpensive carrier (in this case, the microspheres), a two‐step procedure (i.e., separate synthesis of the carrier followed by loading) can be preferred to avoid wastage and loss of the PA during the preparation of the administrable pharmaceutical. For delayed release of pharmaceutical agents, our simple PDMS microspheres may be useful: R6G release into water is slow with only ≈25% release after 200 h. In contrast, release into ethanol is fast and complete upon mixing. We speculate this is primarily a result of the extreme hydrophobicity of PDMS and its poor wettability, given that the solubility of R6G in water is relatively high (≈20 mg mL^−1^).

Improved wettability can be achieved, however, with more polar silicone or copolymeric microspheres, which can be synthesized by either adding an additional component to the precursor solution or using postsynthetic modification of the microsphere surface. Towards this end, we have synthesized three other formulations of microspheres, made from (1) polydiphenyl‐*co*‐polydimethylsiloxane, (2) polytrifluoropropyl‐*co*‐polydimethylsiloxane, and (3) polydimethylsiloxane‐*co*‐poly(propylene oxide‐ethylene oxide) (Figure S8, Supporting Information). Drug delivery studies with these new formulations are underway.

We have presented here a simple, scalable, and continuous process for making micrometer‐sized silicone spheres using ultrasonic spray pyrolysis. Silicones are one of the world's most important and widely used polymers; as such, microspheres made from this material are likely to create novel technologies and new science in a number of disciplines. Prior to the results presented here, the primary limitation to exploring the potential applications of silicone microspheres has been the exceptional difficulty of their synthesis. This USP method overcomes the limitations inherent in past polymerization techniques by isolating the silicone oligomers into micrometer‐sized spray droplets during polymerization, therefore nearly eliminating coalescence and aggregation during microsphere formation. In contrast, this is not possible with traditional emulsion poly­merizations due to the very low surface energy of silicones, which results in only large, polydisperse silicone spheres. The microspheres that result from USP have much smaller diameters and narrower size distributions, compared to products obtained using conventional synthetic methods. Magnetic, fluorescent, and copolymeric silicone microspheres with diameters ranging from <500 nm to ≈2 μm and a relatively narrow size distribution have been produced. These microspheres are easily taken into cells' cytosol, have extremely low cytotoxicity even at a concentration of 100 000 spheres/cell, and have shown potential as drug loading and release materials.

## Supporting information

As a service to our authors and readers, this journal provides supporting information supplied by the authors. Such materials are peer reviewed and may be re‐organized for online delivery, but are not copy‐edited or typeset. Technical support issues arising from supporting information (other than missing files) should be addressed to the authors.

Supplementary
