# Supplementary material for: Magnetic, Fluorescent, and Copolymeric Silicone Microspheres
Source: Adv Sci (Weinh). 2015 May 5;2(6):1500114. doi: 10.1002/advs.201500114 (PMC5115411; doi:10.1002/advs.201500114)
Supplement: Supplementary file 1 — Supplementary [file ADVS-2-0m-s001.pdf]

Copyright WILEY-VCH Verlag GmbH & Co. KGaA, 69469 Weinheim, Germany, 2015.

ADVANCED SCIENCE
Open Access

Supporting Information

for *Adv. Sci.*, DOI: 10.1002/advs.201500114

Magnetic, Fluorescent, and Copolymeric Silicone Microspheres

*Jacqueline M. Rankin, Nitin K. Neelakantan, Kimberly E. Lundberg, Elissa M. Grzincic, Catherine J. Murphy, and Kenneth S. Suslick**

Supporting Information

Magnetic, Fluorescent, and Copolymeric Silicone Microspheres

*Jacqueline M. Rankin, Nitin K. Neelakantan, Kimberly E. Lundberg, Elissa M. Grzincic, Catherine J. Murphy, and Kenneth S. Suslick**

Department of Chemistry, University of Illinois at Urbana-Champaign, 600 S. Matthews Ave. Urbana, IL 61801, USA.
*Correspondence to: E-mail: ksuslick@illinois.edu

Materials and Methods:

Ultrasonic Spray Pyrolysis:

Sylgard 184 polydimethylsiloxane (PDMS) precursor was purchased from Dow Corning as a two-part kit. PDMS microsphere precursor solutions contained the PDMS precursor in a 2:1 base:accelerator ratio. Colloidal Fe_3O_4 nanoparticles (~10 nm) were purchased as a stable suspension under the trade name Magna View Fluid from United Nuclear. The fluid consisted of 5% magnetite, 10% surfactant (oleic acid), and 85% oil carrier by volume. Polydiphenyl-co-polydimethylsiloxane microspheres were synthesized using a precursor solution containing 1.9 v/v % 15-17% diphenylsiloxane-dimethylsiloxane copolymer vinyl terminated (PDV-1625, Gelest Inc.), 0.07 v/v% 25-35% methylhydrosiloxane-dimethylsiloxane copolymer (HMS-301, Gelest Inc.), 0.02 v/v% platinum-divinyltetramethyl-disiloxane complex in xylene (SIP6831.2LC, Gelest Inc.) in hexanes. Polytrifluoropropyl-co-polydimethylsiloxane microspheres were synthesized using a precursor solution containing 1.4 v/v% 35-45% trifluoropropylsiloxane-dimethylsiloxane copolymer vinyl terminated (FMV-4035, Gelest Inc.), 0.7 v/v% HMS-301, 0.02 v/v% SIP6831.2LC in hexanes. Polydimethylsiloxane-co-poly(propylene oxide-ethylene oxide) microspheres were synthesized using a precursor solution containing 1.4 v/v% 30% non-silicone dimethylsiloxane-vinylmethylsiloxane-(propylene oxide-ethylene oxide) block copolymer (DBP-V102, Gelest Inc.), 0.7 v/v% HMS-

301, 0.02 v/v% SIP6831.2LC in toluene. All other reagents were purchased from Sigma Aldrich and used as received without further purification.

As shown in Figure S1, a 1.7 MHz piezoelectric transducer is used to nebulize a precursor solution (e.g., 20 mg/mL PDMS in hexanes) into a mist of micrometer-sized droplets. The aerosol is carried into and through a heated furnace tube via an inert argon stream at 0.4 slpm. The droplets act as micron-sized reaction vessels; the hexanes solvent quickly evaporates and Sylgard 184 precursors, catalyzed by a Pt catalyst, cross-link in the heated furnace (300 °C). The polymerization of PDMS happens very quickly, as the residence time in the furnace is only ~70 seconds, and since each droplet acts as an individual reaction vessel, individual microspheres are produced. The cured PDMS microspheres are collected in ethanol bubblers; ethanol was chosen because it is miscible with hexane, dissolves the uncured PDMS precursors, and easily disperses the cured microspheres. Following collection, the ethanol/microsphere suspension was centrifuged at ~5000 rpm for 60 minutes until the microspheres formed a pellet in the bottom of the centrifuge tube, the ethanol supernatant was decanted, fresh ethanol (~40 mL) was added, and the microspheres were re-dispersed via sonication. This washing protocol was repeated three times; the washed microspheres were suspended in hexanes and stored.

Cell Culture:

Metastatic human breast cancer cells of the MDA-MB-231 cell line (ATCC) were used for microsphere uptake and viability experiments. All live cell work was done in a sterile environment with sterile materials, and live cells were kept at 37°C and 5% CO_2. Cells were cultured in phenol red-free high-glucose DMEM (Corning) with 1 mM sodium pyruvate (Corning), 10% fetal bovine serum (Gemini Bio-Products), 1% penicillin/streptomycin (Corning), 0.1 mM non-essential amino acids (Corning), and 2.5 μg/mL Fungizone (Gibco).

Microsphere Uptake:

20,000 cells (in complete culture medium) were plated into the well of a 35 mm well glass-bottomed culture dish (MatTek Corporation). After incubation for 24 hours, the medium was replaced with 200 μL of the 1.5 mg/mL PDMS microsphere solution (in medium). These samples were incubated for another 24 hours, before washing five times with PBS (phosphate buffered saline solution) to remove free microspheres. Samples were then prepared for imaging, with multiple PBS washes between steps. Cells were first fixed with pre-warmed 4% (in PBS) paraformaldehyde (Sigma) for 15 min. and permeabilized with 0.5% (in PBS) Triton X-100 (Sigma) for 10 min. They were then stained for F-actin with 1:100 fluorescein phalloidin (Molecular Probes, 1 h incubation) and for cell nuclei with 300 nM 4',6-diamidino-2-phenylidole dihydrochloride (DAPI, Molecular Probes, 30 min. incubation). Nile Red-labeled microsphere uptake was then imaged by confocal fluorescence microscopy using a Zeiss LSM 710 confocal microscope.

Cell Viability

20,000 cells (in complete culture medium) were plated into the well of 35 mm well glass-bottomed culture dishes. Three separate cultures were made for each of the tested conditions. After 24 hours of incubation, the medium was replaced with 200 μL of the appropriate concentration of non-fluorescent PDMS microspheres (d_{avg} = 1.15 μm). After a second 24 hour incubation, cells were washed once with PBS and stained using a MarkerGeneTM Live:Dead/Cytotoxicity Assay Kit. The working solution (in PBS) contained 2 μM carboxyfluorescein diacetate for staining live cells, and 4 μM propidium iodide for staining dead cells. Samples were imaged by fluorescence microscopy using a Zeiss Axio Observer Z1 inverted compound microscope. Five random spots in the cell layer were imaged for each sample. Representative images are shown in Figure S5.

Rhodamine 6G (R6G) Loading and Release

PDMS microspheres (1.25 mg/mL; d_{avg} = 1.15 μm) were suspended in a 10 mg/mL solution of R6G in $CHCl_3$. $CHCl_3$ was used as the loading solvent because the USP PDMS microspheres form a stable suspension in $CHCl_3$, R6G is soluble in $CHCl_3$, and $CHCl_3$ swells PDMS.[1] The microsphere/R6G/$CHCl_3$ mixture was put on the rotisserie overnight. The loaded PDMS microspheres were isolated from the $CHCl_3$/R6G solution via centrifugation (3400 rpm, 2 hrs). The microspheres were washed three times with 10 mL cold water. Each washing consisted of adding the water to the centrifuge tube, inverting the tube several times, centrifuging at 3400 rpm for 30 minutes, and immediately removing the supernatant using a pipette. After the final washing, the spheres were suspended in 10 mL of phosphate buffered saline (PBS, Thermo Scientific, w/o calcium or magnesium) to a concentration of 0.85 mg loaded microspheres/mL. This suspension was put on the rotisserie and kept at 37 °C for the release experiments. At each time point (0 minutes, 30 minutes, 1 hour, 1.5 hrs, 3 hrs, 5hrs, 10 hrs, 25 hrs, 50 hrs, 75 hrs, and 100 hrs), 2.0 mL of the PBS solution was removed and analyzed with UV/VIS (Varian Cary 5G UV-VIS-NIR spectrophotometer); 2.0 mL of fresh PBS was added to the suspended microspheres immediately. To quantify the total R6G loaded into the PDMS microspheres, loaded microspheres were suspended in ethanol, the solution isolated, and the absorbance at 525 nm determined using UV/VIS. The calibration curve used for these experiments is given in Figure S6.

Characterization:

SEM:

Generally, scanning electron micrographs were obtained on a JEOL 7000F instrument operating at 10 kV with a medium probe current and a working distance of 10 mm. Samples were prepared by freeze drying a suspension of PDMS microspheres in methylcyclohexane on a Si wafer. Samples were mounted to the holder via carbon tape and sputter coated with

approximately 10 nm of Au/Pd prior to analysis to prevent surface charging. Size distribution analysis was performed using Image J software.

TGA:

Thermogravimetric analysis of the PDMS microspheres (Figure S2) was obtained using a TA Instrument Q50 TGA. A 2.21 mg sample was heated from 20 °C to 300 °C at a rate of 20 °C/min and 300 °C to 750 °C at a rate of 10 °C/min under air (60 mL/min). As is expected, the TGA of the PDMS microspheres matches TGA of bulk PDMS.[2] Mass loss before 200 °C is minimal, mass loss between 200 °C and ~400 °C is attributed to the formation of volatile cyclosiloxanes, and loss above 400 °C is attributed to oxidation into silica and the formation of various silicon/carbon species.[3]

IR:

Infrared spectroscopic data was collected using a Perkin Elmer Spectrum 100 FT-IR instrument using a diamond/ZnSe attenuated total reflection (ATR) accessory.

Raman:

Raman was collected using a Nanophoton Raman-11 laser Raman microscope with a 532 nm laser. Samples were prepared by freeze-drying a suspension of PDMS microspheres in methylcyclohexane on a Si wafer. The peak at 2906 cm^{-1}, characteristic of a C-H stretching mode in PDMS,[4] was used to map the regions of PDMS on the sample.

UV/VIS Diffuse Reflectance:

A Prime-X™ back-thinned CCD array spectrometer (2.5 nm resolution), Deuterium (30 W)- Tungsten (5 W) light source, and fiber optic reflectance probe (Seven 400 μm illuminates and one 600 μm read fiber with a read diameter of 1 mm) was used to collect the UV/VIS

diffuse reflectance measurements. Microspheres were analyzed using PVDF as a white background. Data was smoothed using a 20 point Savitzky-Golay smoothing filter and converted to a pseudo-absorbance using the Kubelka-Munk equation. Data from 485.35-487.76 nm and 654.08-657-89 nm was removed prior to analysis; these regions show distortion due to hydrogen emission lines characteristic of the deuterium light source.

Fluorescence Imaging:

Fluorescence imaging was done using a Zeiss Axiovert 200M inverted research-grade microscope with a 41039 special yellow filter. Samples were prepared by drop-casting a suspension of the microspheres in hexane on a glass cover slip with immediate heating at 70 °C. The non-fluorescent microspheres were also imaged, and as expected showed no measureable fluorescence (not shown).

EDS:

Energy dispersive spectrometry (EDS) line scans were obtained using a JEOL 7000F instrument equipped with a Thermo Electron EDS microanalysis system operating at 10 kV with a high probe current and a working distance of 10 mm. Samples were prepared by freeze drying a suspension of PDMS microspheres in methylcyclohexane on Cu foil. Samples were mounted to the holder via carbon tape and sputter coated with approximately 10 nm of Au/Pd prior to analysis to prevent surface charging.

TEM:

Transmission electron micrographs were obtained on a JEOL 2100 cryo microscope operating at 200 kV and equipped with a Gatan MatScan1kx1k progressive scan CCD camera. Samples were prepared by freeze-drying a suspension of PDMS microspheres in methylcyclohexane on a lacy formvar/carbon 200 mesh copper grid (Ted Pella, #01881-F).

Table S1. Small molecule octanol/water partition coefficients (LogP).

	LogP	Use
**Rhodamine 6G**[a]	2.67 [5]	Fluorescent dye
**Ketoprofen**	2.77 [6]	NSAID
**Diltiazem**	2.79 [7]	Calcium channel blocker
**Warfarin**	2.60 [7]	Anticoagulant

[a]Used in this study

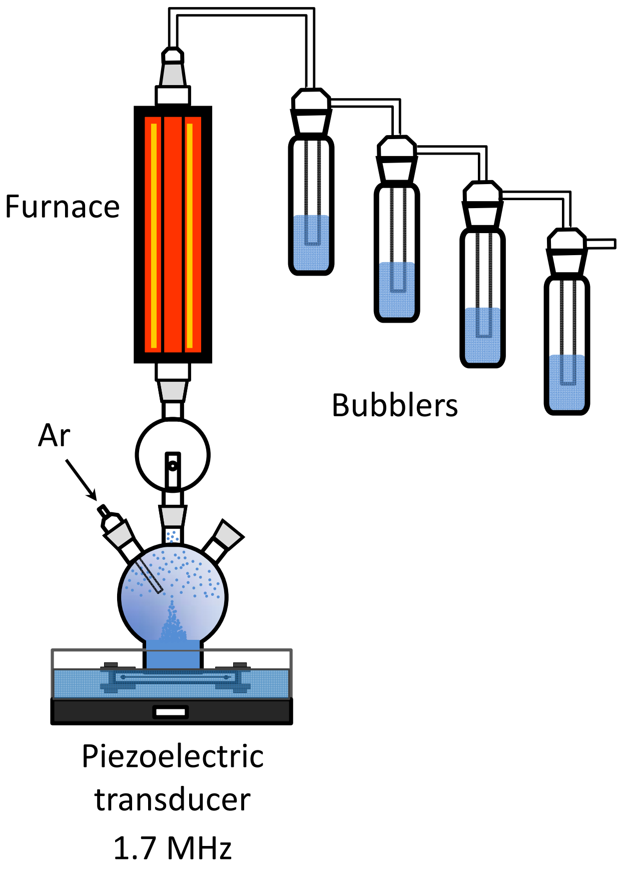

Figure S1: Ultrasonic spray pyrolysis (USP) experimental setup.

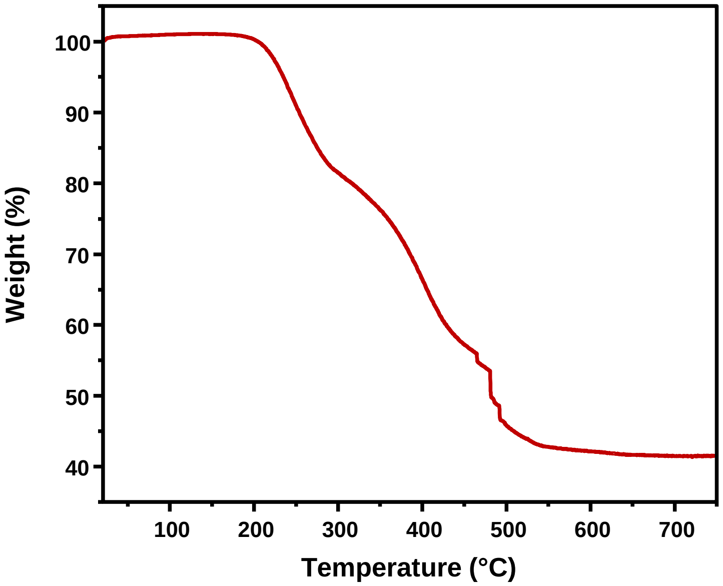

Figure S2: Thermogravimetric analysis of polydimethylsiloxane microspheres. The TGA of the PDMS microspheres matches TGA of bulk PDMS.[2] Mass loss before 200 °C is minimal, mass loss between 200 °C and ~400 °C is attributed to the formation of volatile cyclosiloxanes, and loss above 400 °C is attributed to oxidation into silica and the formation of various silicon/carbon species.[3]

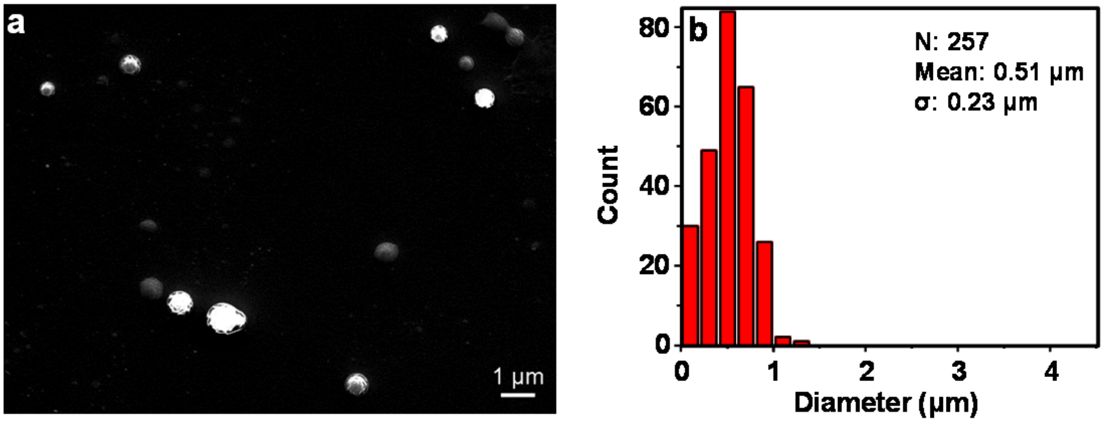

Figure S3: (a) Scanning electron micrograph and (b) size distribution of polydimethylsiloxane microspheres obtained with a 1 mg/mL PDMS in hexane precursor solution.

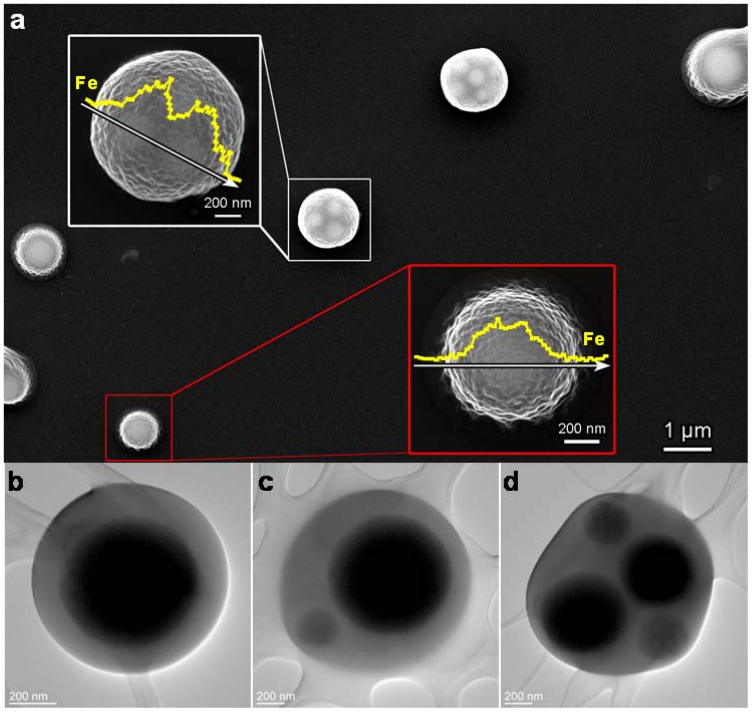

Figure S4: (a) Scanning electron micrograph of magnetic polydimethylsiloxane microspheres (accelerating voltage of 20 kV). Insets show expanded view of individual microspheres with EDS trace for iron shown in yellow. (b-d) Transmission electron micrographs of magnetic PDMS microspheres showing one, two, and many iron cores.

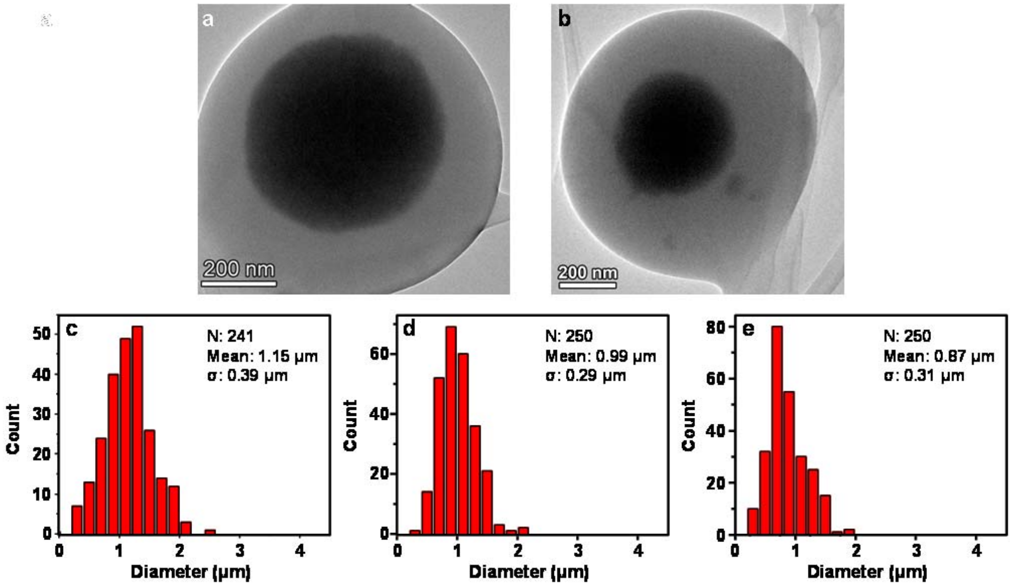

Figure S5: (a, b) Transmission electron micrographs of a magnetic polydimethylsiloxane (PDMS) microsphere obtained with (a) a precursor solution containing a 1:1 v/v ratio of PDMS (20 mg/mL hexane) to Magnafluid and (b) a precursor solution containing a 3:1 v/v ratio of PDMS (20 mg/mL hexane) to Magnafluid. (c-e) Size distribution of PDMS microspheres obtained with (c) a 20 mg/mL PDMS in hexane precursor solution, (d) a precursor solution containing a 1:1 v/v ratio of PDMS (20 mg/mL hexane) to Magnafluid, and (e) a precursor solution containing a 3:1 v/v ratio of PDMS (20 mg/mL hexane) to Magnafluid.

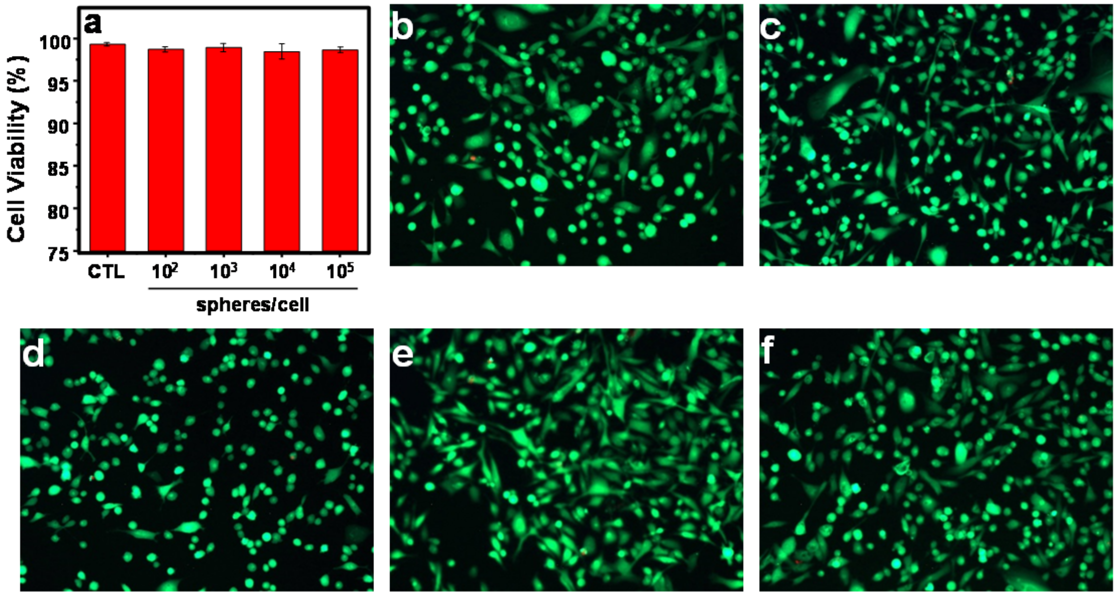

Figure S6: Ultrasonic spray pyrolysis polydimethylsiloxane (PDMS) microsphere cytotoxicity. (a) Cytotoxicity study; no statistically significant differences in cell viability are seen among the control and concentrations of PDMS microspheres ranging from 100 to 100,000 spheres/cell. (b-f) Representative fluorescence images of stained cells from polydimethylsiloxane microsphere viability experiments. (b) Control. (c) 100 spheres/cell. (d) 1000 spheres/cell. (e) 10,000 spheres/cell. (f) 100,000 spheres/cell. Cells that are stained green are alive, cells that are stained red are dead.

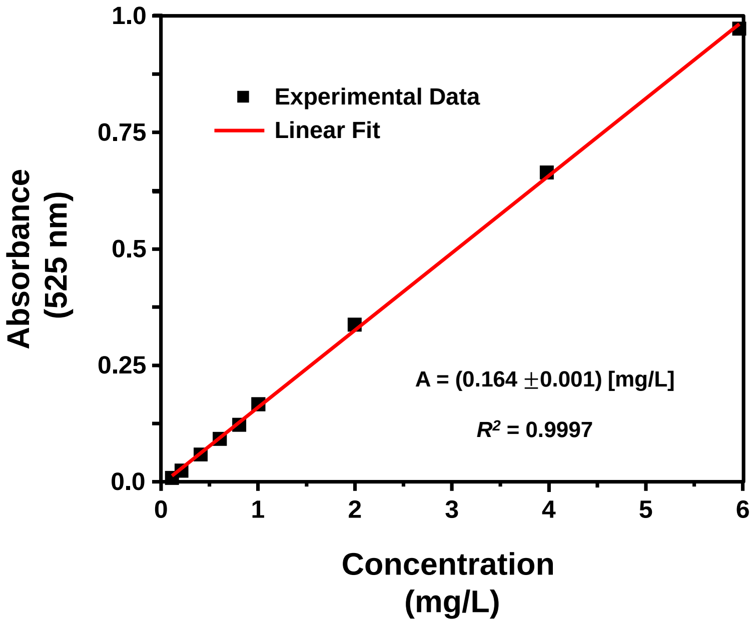

Figure S7: Calibration curve for Rhodamine 6G in phosphate buffered saline.

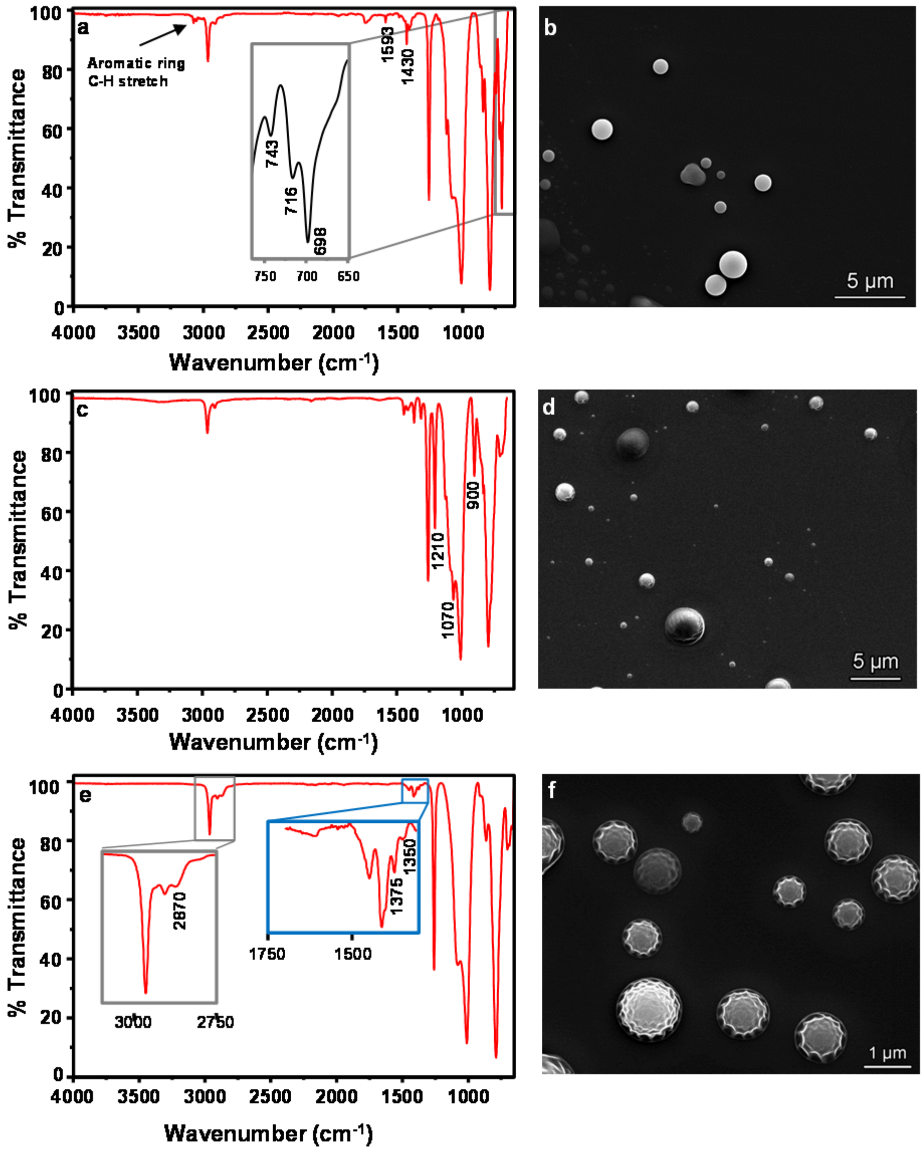

Figure S8: Copolymeric silicone microspheres fabricated using ultrasonic spray pyrolysis. (a & b) ATR-FTIR and SEM, respectively, of polydiphenyl-co-polydimethylsiloxane microspheres. Peaks in (a) corresponding to the polydiphenylsiloxane chains are labeled; defining peaks include sharp bands at 1600 and 1430 cm^{-1} and a set of three bands in the fingerprint region at ~ 740, 720 and 700 cm^{-1} that have increasing band intensity with decreasing wavenumber.[8] (c & d) ATR-FTIR and SEM, respectively, of polytrifluoropropyl-co-polydimethylsiloxane microspheres. Peaks in (c) corresponding to the polytrifluoropropylsiloxyl chains are labeled; defining peaks include a peak at 1210, 1070, and 900 cm^{-1}.[8] (e & f) ATR-FTIR and SEM, respectively, of polydimethylsiloxane-co-poly(propylene oxide-ethylene oxide) microspheres. Peaks in (e) corresponding to the poly(propylene oxide-ethylene oxide) regions are labeled; the stretches at 2870, 1375, and the shoulder at 1350 cm^{-1}, which can be attributed to, respectively, a CH_3 stretching mode, the symmetrical bending vibration of the methyl group, and the wagging vibration of the methylene group of the poly(propylene oxide-ethylene oxide) chains.[9] The FT-IR spectra (a,c,e) show all characteristic peaks expected for polydimethylsiloxane.

[1] J. N. Lee, C. Park, G. M. Whitesides, *Anal. Chem.* **2003**, *75*, 6544.

[2] R. T. Johnson, R. M. Biefeld, J. A. Sayre, *Polym. Eng. Sci.* **1984**, *24*, 435.

[3] G. Camino, S. M. Lomakin, M. Lazzari, *Polymer* **2001**, *42*, 2395.

[4] F. C. Adam, K. Khashayar, R. S. Paul, M. Arnan, K. z. Kourosh, *Chem. Soc. Rev.* **2013**, *42*, 5880.

[5] J. Wang, N. Douville, S. Takayama, M. ElSayed, *Ann. Biomed. Eng.* **2012**, *40*, 1862.

[6] X. Liu, H. Hefesha, G. Scriba, A. Fahr, *Helv. Chim. Acta* **2008**, *91*, 1505.

[7] A. Kokate, X. Li, P. Williams, P. Singh, B. Jasti, *Pharm. Res.* **2009**, *26*, 1130.

[8] B. Arkles, Larson, G. L., Ed., *Silicon Compounds: Silanes & Silicones, 5000A, 3rd ed*, Gelest Inc, Morrisville, PA. **2013**.

[9] G. Meszlényi, M. Sipos, É. Juhász, M. Eröss-Lelkes, G. Poszmik, *Acta Physica Hungarica* **1988**, *63*, 137.
